# Supplementary material for: Identification of photoperiod-induced specific miRNAs in the adrenal glands of Sunite sheep (Ovis aries)
Source: Front Vet Sci. 2022 Jul 22;9:888207. doi: 10.3389/fvets.2022.888207 (PMC9354845; doi:10.3389/fvets.2022.888207)
Supplement: Supplementary file 1 [file Data_Sheet_1.ZIP › Supplementary file/miRDeep2 file.zip!/miRDeep2 file/Know_Structure/LP42A3/oar-mir-10b.pdf]

miRBase precursor : oar-mir-10b
Total read count : 881878
oar-miR-10b read count : 878686
remaining reads : 3192

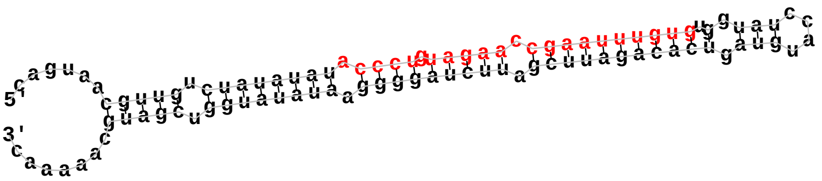

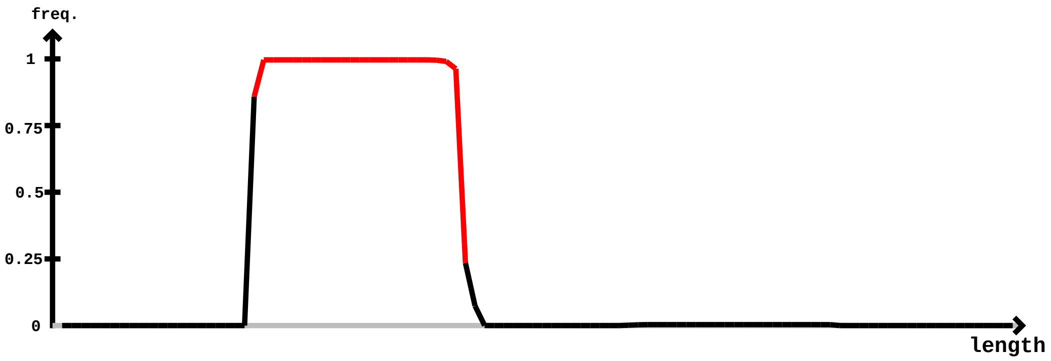


```
                   oar-miR-10b
5'- caguaacguugucuauauauacccuguagaaccgaauuugugugguauccauguagucacagauucgauucuaggggaauauauggucgaugcaaaaac   -3'    exp
    ......(((((.((((((((.((((.(((((.((((((((((.(.(((....))).))))))))))).))))))))).)))))))).))))).......   reads      mm         sample
    .................uauacccuguagaaccgaauuugu..........................................................   1          0          seq
    ..................auacccuguagaaccgaauuug...........................................................   1          0          seq
    ..................auacccuguagaaccgaauuugu..........................................................   20         0          seq
    ...................uacccuguagaaccgaa...............................................................   14         0          seq
    ...................uacccuguagaaccgaau..............................................................   65         0          seq
    ...................uacccuguagaaccgaauu.............................................................   849        0          seq
    ...................uacccuguagaaccgaauuu............................................................   3749       0          seq
    ...................uacccuguagaaccgaauuug...........................................................   22531      0          seq
    ...................uacccuguagaaccgaauuugu..........................................................   603713     0          seq
    ...................uacccuguagaaccgaauuugug.........................................................   102009     0          seq
    ...................uacccuguagaaccgaauuugugu........................................................   24399      0          seq
    ...................uacccuguagaaccgaauuugugug.......................................................   24         0          seq
    ...................uacccuguagaaccgaauuugugugg......................................................   3          0          seq
    ...................uacccuguagaaccgaauuuguguggu.....................................................   2          0          seq
    ....................acccuguagaaccgaau..............................................................   4          0          seq
    ....................acccuguagaaccgaauu.............................................................   69         0          seq
    ....................acccuguagaaccgaauuu............................................................   434        0          seq
    ....................acccuguagaaccgaCuuu............................................................   1          1          seq
    ....................acccuguagaaccgaauuug...........................................................   1755       0          seq
    ....................acccuguagaaccgaauuugu..........................................................   38022      0          seq
    ....................acccuguagaaccgaauuugug.........................................................   40332      0          seq
    ....................acccuguagaaccgaauuugugu........................................................   40468      0          seq
    ....................acccuguagaaccgaauuugugug.......................................................   107        0          seq
    ....................acccuguagaaccgaauuugugugg......................................................   8          0          seq
    ....................acccuguagaaccgaauuuguguggu.....................................................   2          0          seq
    .....................cccuguagaaccgaauuug...........................................................   1          0          seq
    .....................cccuguagaaccgaauuugu..........................................................   9          0          seq
    .....................cccuguagaaccgaauuugug.........................................................   12         0          seq
    .....................cccuguagaaccgaauuugugu........................................................   52         0          seq
    ......................ccuguagaaccgaauuugu..........................................................   9          0          seq
    ......................ccuguagaaccgaauuugug.........................................................   5          0          seq
    ......................ccuguagaaccgaauuugugu........................................................   5          0          seq
    .......................cuguagaaccgaauuugu..........................................................   4          0          seq
    .......................cuguagaaccgaauuugug.........................................................   2          0          seq
    .......................cuguagaaccgaauuugugu........................................................   5          0          seq
```

oar-miR-10b

```
caguaacguugucuauauauacccuguagaaccgaauuugugugguauccauguagucacagauucgauucuaggggaauauauggucgaugcaaaaac
.......................cuguagaaccgaauuugugugguauc..................................................    1       0       seq
........................uguagaaccgaauuugu..........................................................    1       0       seq
........................................................ucacagauucgauucuagggga.....................    1       0       seq
........................................................ucacagauucgauucuaggggaa....................    1       0       seq
........................................................ucacagauucgauucuaggggaau...................    1       0       seq
.........................................................cacagauucgauucuagggga.....................    3       0       seq
.........................................................cacagauucgauucuaggggaa....................    4       0       seq
.........................................................cacagauucgauucuaggggaau...................    12      0       seq
..........................................................acagauucgauucuagggga.....................    35      0       seq
..........................................................acagauucgauucuaggggaa....................    50      0       seq
..........................................................acagauucgauucuaggggaau...................    1127    0       seq
..........................................................acagauucgauucuaggggaaua..................    6       0       seq
...........................................................cagauucgauucuagggga.....................    25      0       seq
...........................................................cagauucgauucuaggggaa....................    32      0       seq
...........................................................cagauucgauucuaggggaau...................    1203    0       seq
...........................................................cagauucgauucuaggggaaua..................    13      0       seq
...........................................................cagauucgauucuaggggaauau.................    4       0       seq
............................................................agauucgauucuagggga.....................    9       0       seq
............................................................agauucgauucuaggggaa....................    11      0       seq
............................................................agauucgauucuaggggaau...................    579     0       seq
............................................................agauucgauucuaggggaaua..................    38      0       seq
............................................................agauucgauucuaggggaauau.................    31      0       seq
............................................................agauucgauucuaggggaauaua................    2       0       seq
.............................................................gauucgauucuaggggaau...................    2       0       seq
.............................................................gauucgauucuaggggaauau.................    1       0       seq
```
